# CLN3 Deficient Cells Display Defects in the ARF1-Cdc42 Pathway and Actin-Dependent Events

**DOI:** 10.1371/journal.pone.0096647

**Published:** 2014-05-02

**Authors:** Mark L. Schultz, Luis Tecedor, Colleen S. Stein, Mark A. Stamnes, Beverly L. Davidson

**Affiliations:** 1 Program of Molecular and Cellular Biology, University of Iowa, Iowa City, Iowa, United States of America; 2 Department of Internal Medicine, University of Iowa, Iowa City, Iowa, United States of America; 3 Department of Molecular Physiology and Biophysics, Iowa City, Iowa, United States of America; 4 Department of Neurology, Iowa City, Iowa, United States of America; National Eye Institute, United States of America

## Abstract

Juvenile Batten disease (juvenile neuronal ceroid lipofuscinosis, JNCL) is a devastating neurodegenerative disease caused by mutations in CLN3, a protein of undefined function. Cell lines derived from patients or mice with CLN3 deficiency have impairments in actin-regulated processes such as endocytosis, autophagy, vesicular trafficking, and cell migration. Here we demonstrate the small GTPase Cdc42 is misregulated in the absence of CLN3, and thus may be a common link to multiple cellular defects. We discover that active Cdc42 (Cdc42-GTP) is elevated in endothelial cells from CLN3 deficient mouse brain, and correlates with enhanced PAK-1 phosphorylation, LIMK membrane recruitment, and altered actin-driven events. We also demonstrate dramatically reduced plasma membrane recruitment of the Cdc42 GTPase activating protein, ARHGAP21. In line with this, GTP-loaded ARF1, an effector of ARHGAP21 recruitment, is depressed. Together these data implicate misregulated ARF1-Cdc42 signaling as a central defect in JNCL cells, which in-turn impairs various cell functions. Furthermore our findings support concerted action of ARF1, ARHGAP21, and Cdc42 to regulate fluid phase endocytosis in mammalian cells. The ARF1-Cdc42 pathway presents a promising new avenue for JNCL therapeutic development.

## Introduction

Juvenile neuronal ceroid lipofuscinosis (JNCL), caused by mutations in *CLN3*, is a lysosomal storage disease (LSD) with an incidence reaching 1∶25,000 in northern European countries [1]. JNCL patients develop visual symptoms at 4–7 years of age, leading rapidly to blindness. This is followed by progressive seizures, physical and mental decline, and most affected individuals succumb to disease by the second or third decade of life [2], [3]. By magnetic resonance imaging, JNCL patients have progressive volume loss in most brain regions [4], [5], [6]. While intracellular accumulation of autofluorescent material is well documented and the natural history described, the mechanisms by which CLN3 deficiency induces pathogenesis in JNCL is not understood. Earlier work showed that autoantibodies against central nervous system proteins were present in JNCL mouse models and patient blood [7], [8], [9]. This could reflect blood brain barrier (BBB) compromise. In a knock-in mouse model for CLN3, wherein the bacterial β-galactosidase reporter was inserted into the endogenous *CLN3* locus, reporter expression was robust in brain endothelial cells [10]. In addition, patient endothelial cells are laden with the characteristic storage inclusions [11], [12]. This suggests that CLN3 is important for brain endothelial cell function and integrity. Using the CLN3 null reporter mouse we have shown multiple defects in intracellular membrane dynamics and protein trafficking both *in vivo* and *in vitro* [13], although the molecular basis for the impact of CLN3 on these processes is not known.

CLN3 is a 438 amino acid protein with six predicted transmembrane domains [14]. Notably, a lack of sensitive antibodies precludes reliable detection of endogenous CLN3 *in situ*. *In vitro* analyses using overexpression systems has localized CLN3 to the Golgi, plasma membrane, synaptosomes, late endosomes, and lysosomes [3], [14]. CLN3 deficiency is reported to cause defects in cell motility [15], Golgi antero- and retrograde trafficking, lysosomal pH, autophagy, lipid metabolism or transport, and endocytosis [3], [13].

Impaired endocytosis is a consistent observation in CLN3-deficient cells including yeast, mouse neurons and endothelial cells, and patient fibroblasts [13], [16], [17], [18], [19], [20]. Here, we find that fluid phase endocytosis is also impaired in brain microvascular endothelial cells. Fluid-phase endocytosis relies heavily on the actin cytoskeleton network, and multiple groups have found alterations in the actin cytoskeleton or actin binding proteins [15], [16]. However, how the absence of CLN3 impairs this network remains unknown.

**Figure 1:**
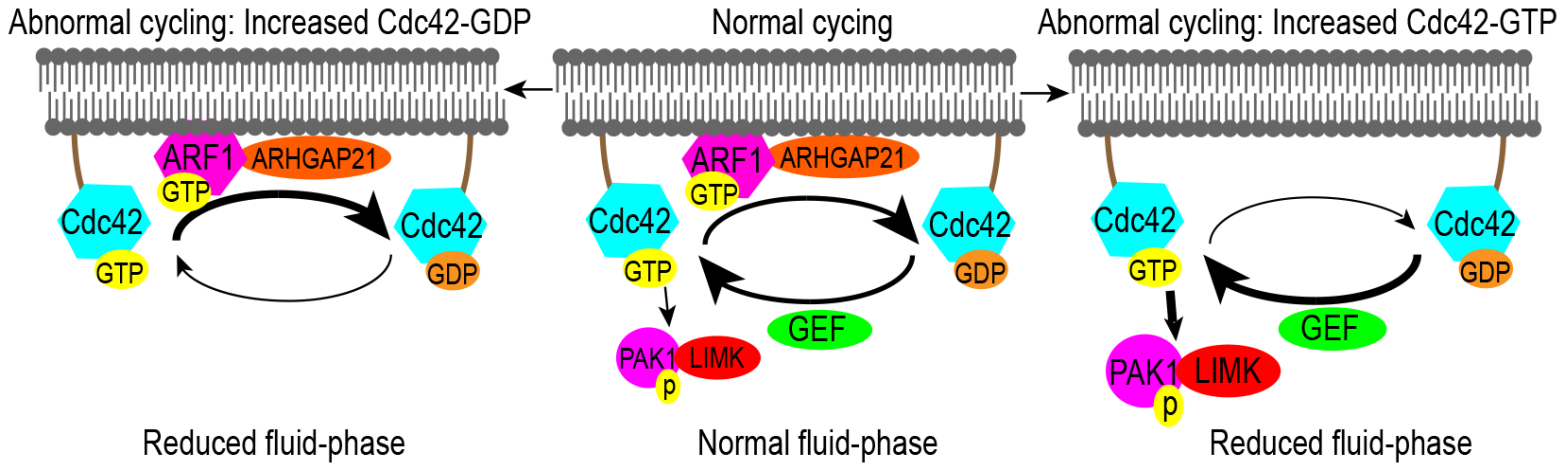
The role of Cdc42 GTP to GDP cycling in fluid phase endocytosis. Fluid-phase endocytosis requires Cdc42 cycling from the GTP to the GDP bound state, which is controlled in part by GAPs and GEFs, and upstream of that, ARF1. Defects in Cdc42 cycling negatively influence endocytosis. In the absence of CLN3, faulty recruitment or function of a regulator (for example ARF1, GAP or GEF) can create an imbalance toward either Cdc42-GDP (left) or Cdc42-GTP (right), which would in turn impair endocytosis.

The small GTPase Cdc42 regulates sequential synthesis and break down of actin allowing fluid-phase uptake to occur [21], [22], [23], [24]. To accomplish this Cdc42 cycles from an active GTP-bound to an inactive GDP-bound state [25]. In the GTP-bound state Cdc42 binds to and subsequently activates target proteins, initiating scaffolding-protein recruitment and signal induction, ultimately triggering actin polymerization. Actin filament formation facilitates inward budding, scission, and the early vesicle transport events of endocytosis. Actin disassembly is necessary for continuous rounds of endocytosis, and dynamic Cdc42 cycling is critical for orchestrating polymerization/depolymerization events. Notably, if Cdc42 is constrained in either the GTP or GDP loaded state, fluid-phase uptake is inhibited [26], [27]. Based on the requirement for Cdc42 cycling, CLN3 deficiency could impair fluid phase endocytosis by either enhancing or reducing Cdc42 pathway activation as shown in Fig. 1.

Regulation of Cdc42 cycling is executed by GTPase activating proteins (GAPs), which increase GTP hydrolysis, and guanine nucleotide exchange factors (GEFs) which facilitate removal of the tightly bound GDP, allowing GTP reloading (Fig. 1) [25]. Recruitment of the GAP, ARHGAP21 (also known as ARHGAP10), to the plasma membrane is essential for modulating the plasma membrane activity of Cdc42 [27]; ARHGAP21 knock-down induces increased Cdc42 membrane localization, filopodia formation, actin filament disorganization, and inhibition of fluid-phase endocytosis [27]. By co-immunoprecipitation [28] and crystallography studies [29] ARHGAP21 interacts with and is regulated by GTP-loaded ARF1, another small GTPase. We hypothesized that misregulation of Cdc42 underlies endocytic and other actin-based defects in CLN3 deficient cells. To test this, we assessed Cdc42 activity and examined factors that function upstream and downstream of Cdc42 (Fig. 1). Herein we show that GTP-loaded Cdc42 is elevated in CLN3 null MBEC, with reduced GTP-loaded ARF1 and impaired plasma recruitment of ARHGAP21.

## Results

### Altered fluid-phase endocytosis and increased Cdc42-GTP in CLN3-deficient MBECs

**Figure 2:**
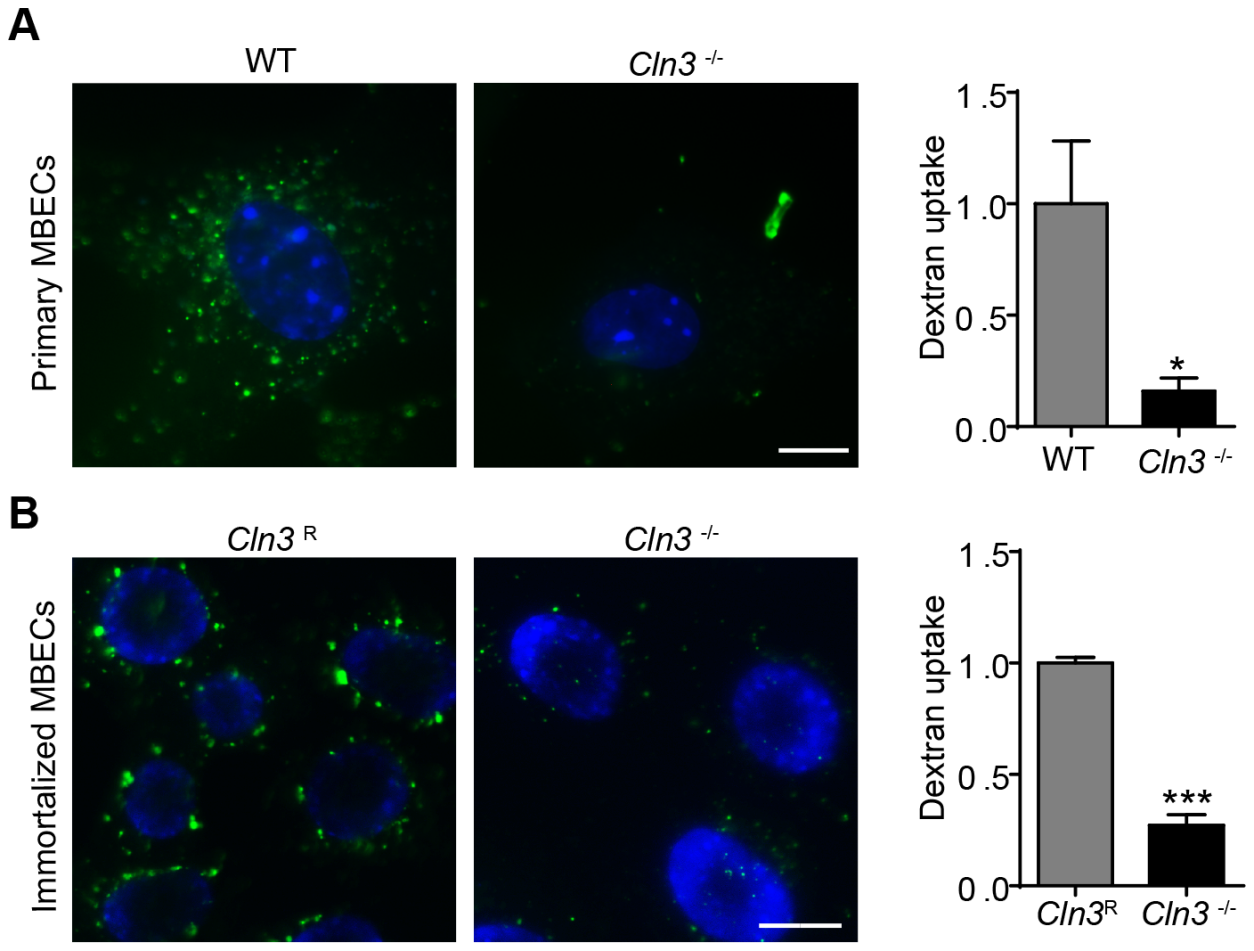
Fluid-phase endocytosis is impaired in CLN3-null MBECs. Primary (A) and immortalized (B) MBECs of the indicated genotypes were incubated with Hoechst 33342 to label cell nuclei (*blue*) followed by incubation in Alexa 488 conjugated dextran (*green*). A488-Dextran uptake was evaluated by fluorescence microscopy and intensity quantified using ImageJ. Data are the mean of three independent experiments. Error bars ± s.e.m. (t-test, *, p<0.05, ***, p<0.001). Scale bars are 10 µm (A) and 20 µm (B).

Decreased levels of fluid-phase endocytosis are widely reported in CLN3 mutant cells [16], [17], [18], [19], [20], but the underlying molecular mechanism has not been investigated, nor has this phenotype been reported in brain endothelial cells. To address this we used primary cultures of MBECs from wildtype (WT) and our CLN3-null (*Cln3* ^lacZ/lacZ^) mice and measured fluid-phase endocytosis by incubating them with fluorescently-labeled dextran. Primary *Cln3* ^−/−^ cells display poor fluid-phase uptake, with quantification indicating 84% decrease relative to WT cells (Fig. 2A). Immortalized MBECs generated from *Cln3* ^−/−^ mice similarly display poor dextran uptake, and stable re-introduction of *Cln3* (*Cln3* ^R^) restores fluid-phase endocytosis (Fig. 2B).

**Figure 3:**
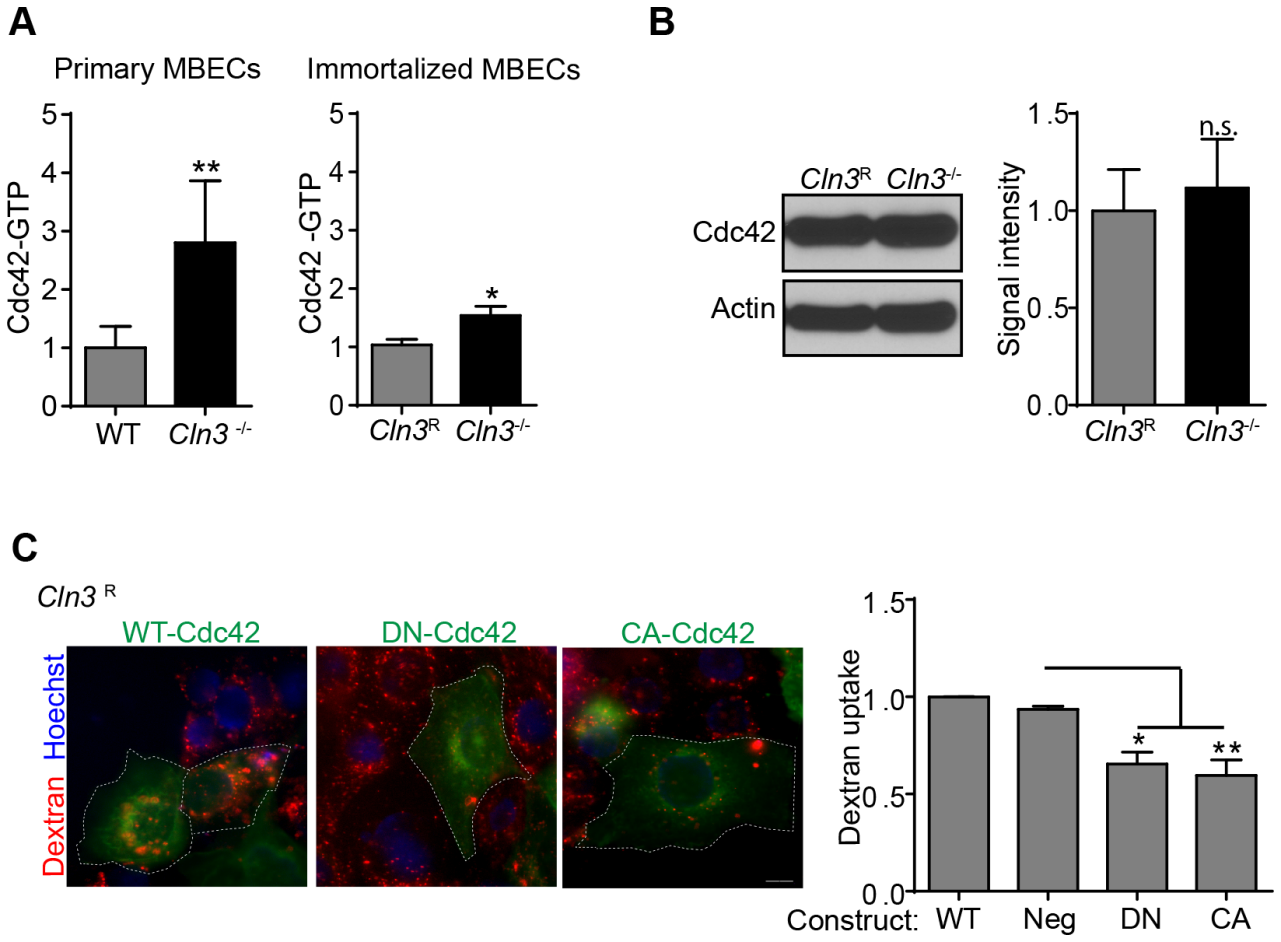
Cdc42-GTP is elevated in CLN3-null MBECs. (A) Cdc42-GTP activity was quantified from primary (WT and *Cln3* ^−/−^) or immortalized (*Cln3* ^R^ and *Cln3* ^−/−^) MBEC lysates. (B) Total Cdc42 protein levels were quantified by western blot, expressed as band intensity normalized to the actin loading control. (C) *Cln3* ^R^ MBECs were transfected with WT-Cdc42-GFP, GFP (negative control), dominant negative (DN)-Cdc42-GFP, or constitutively active (CA)-Cdc42-GFP constructs. Endocytosis of Rhodamine conjugated dextran (*red*) was imaged by epifluorescence and quantified in transfected cells (*green*). Data represent the mean of four (A, B) and three (C) independent experiments. Error bars ± s.e.m. ((A,B) t-test, (C) 1-way ANOVA with Tukey post-hoc, *, p<0.05, **, p<0.005, n.s. =  not significant). (A) Scale bar is 10 µm. Dashed lines indicate the outline of transfected cells.

Unlike clathrin or caveolar endocytosis, fluid-phase endocytosis does not utilize coat proteins to induce vesicle formation, but instead relies on actin-dependent events controlled by the small GTPase, Cdc42 [21]. We assessed whether endogenous levels of GTP-loaded Cdc42 were affected by CLN3 loss. Interestingly, quantification revealed elevated Cdc42-GTP in primary *Cln3* ^−/−^ MBECs compared to WT (Fig. 3A). Increased Cdc42-GTP was also observed, but not as pronounced, in immortalized *Cln3* ^−/−^ relative to *Cln3* ^R^ MBECs (Fig. 3A). To determine whether elevated Cdc42-GFP is a consequence of an overall increase in Cdc42 protein levels, we quantified total Cdc42 levels by western blot. These data revealed no differences in protein expression between *Cln3* ^R^ and *Cln3* ^−/−^ MBECs (Fig. 3B). Thus, increased Cdc42-GTP, in the setting of CLN3 deficiency, represents impaired GTP-to-GDP cycling, which in-turn could explain the endocytosis block. Based on the extent of Cdc42-GFP elevation, one might expect a greater disparity between the primary and immortalized cells with respect to the endocytic defect. We speculate that immortalization likely alters various pathways that involve Cdc42, but that the pool of Cdc42 devoted to regulating endocytosis may be similar between primary and immortalized cells. Nevertheless, our data support defective Cdc42 cycling proximal to impaired fluid phase endocytosis in both primary and immortalized CLN3-null MBEC.

To confirm that Cdc42 cycling is required for efficient fluid-phase uptake in brain endothelia, MBECs were transfected with wildtype (WT), dominant negative (DN), or constitutively active (CA) Cdc42 expressing plasmids and fluid-phase uptake was quantified. We found that overexpression of either CA or DN forms significantly reduced dextran uptake compared to transfection with WT Cdc42 (Fig. 3C). This is consistent with studies from other groups [26], [27], and illustrates the influence of Cdc42 dynamics on this pathway in MBECs.

### CLN3 loss correlates with increased P-PAK-1 and LIMK membrane recruitment

**Figure 4:**
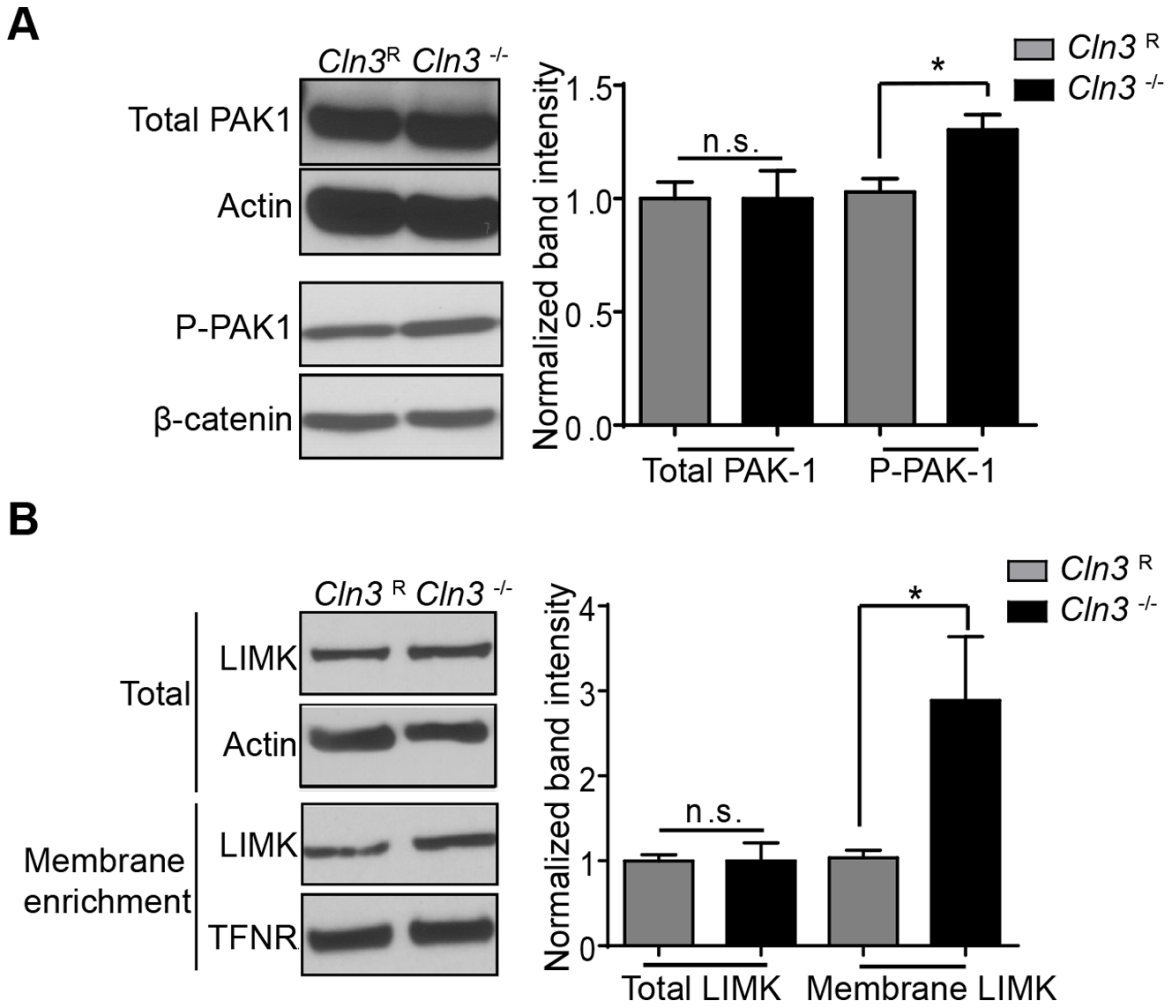
PAK-1 phosphorylation and LIMK recruitment are elevated in CLN3-null MBECs. (A) Total cell lysates or (B) membrane fractions were analyzed by western blot for PAK-1, P-PAK-1, and LIMK. Actin and β-catenin were used as total lysate loading controls, and transferrin receptor (TFNR) as the loading control for membrane enriched samples. Normalized band intensity was calculated as in Fig. 3. Results are the mean of three independent experiments. Error bars represent ± s.e.m. (t-test, *, p<0.05).

Cdc42-GTP induces a well-established signaling cascade that recruits proteins to membranes. Cdc42-GTP activates p21 protein activated kinase 1 (PAK-1), resulting in PAK-1 phosphorylation (P-PAK-1) and LIM kinase domain 1 (LIMK) activation initiating actin polymerization. To investigate downstream components of Cdc42 activation, namely levels of P-PAK-1, *Cln3* ^R^ and *Cln3* ^−/−^ lysates were assessed for P-PAK-1 levels by western blot. Although total amounts of PAK-1 protein were the same in *Cln3* ^R^ and *Cln3* ^−/−^ MBECs, *Cln3* ^−/−^ cells have increased levels of P-PAK-1 (Fig. 4A), consistent with amplified Cdc42-GTP activity.

To quantify LIMK1 recruitment to membranes, total lysates and membrane-enriched lysates were analyzed for LIMK1 by western blot. Similar to P-PAK-1, total LIMK1 levels were equivalent in *Cln3* ^−/−^ and *Cln3* ^R^ preparations, but there was increased LIMK recruitment in *Cln3* ^−/−^ membranes relative to *Cln3* ^R^ controls (Fig. 4B). These findings show that in the absence of CLN3, there is elevated activation of the Cdc42 protein and its downstream effectors.

### Filopodia formation and cell migration are abnormal in *Cln3* ^−/−^ cells

**Figure 5:**
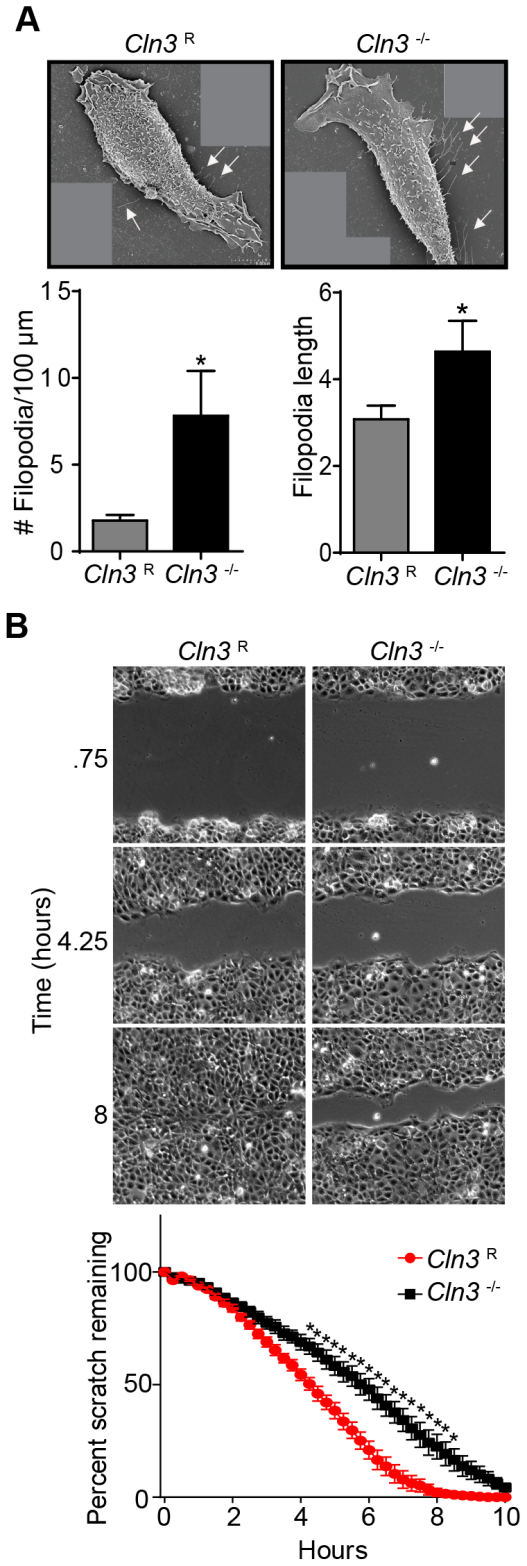
*Cln3* ^−/−^ MBECs have defects in actin dependent processes. (A) SEM images of immortalized MBECs of the indicated genotypes were taken, compiled, and filopodial length and number was measured. *White arrows* indicate filopodia. Filopodia were counted on 32 *Cln3* ^R^ and 36 *Cln3* ^−/−^ MBECs. (B) A scratch was made across a confluent monolayer of cells and migration was assessed via live cell microscopy and quantified. Representative images from three time-points are shown. The graph shows data from three independent experiments ± s.e.m. ((A) t-test, (B) 2-way ANOVA with Bonferroni post-hoc correction, *, p<0.05). Scale bars are 5 µm (A) or 100 µm (B).

Due to the importance of Cdc42 in actin polymerization, we next analyzed actin distribution and morphology in *Cln3* ^R^ and *Cln3* ^−/−^ MBECs using confocal microscopy. There was altered actin staining, and we noted increased filopodial formation in *Cln3* ^−/−^ MBECs (Fig. S1). We quantified filopodia number per length of plasma membrane by scanning electron microscopy (SEM) and discovered that *Cln3* ^−/−^ cells have 4-fold more filopodia compared to *Cln3* ^R^ cells (Fig. 5A). Moreover, the average filopodia length was increased by 50% for *Cln3* ^−/−^ relative to *Cln3* ^R^ cells (Fig. 5A).

In addition to its role in filopodial formation, Cdc42 is involved in establishing cell polarity, and Cdc42 cycling is necessary for polarized cell movement. Live cell microscopy of *Cln3* ^−/−^ and *Cln3* ^R^ MBECs showed that *Cln3* ^−/−^ MBECs are delayed in their ability to migrate into a gap in the monolayer, indicative of impaired polarized cell motility (Fig. 5B). Cell polarity defects have been described in *btn1* ^−/−^ yeast (CLN3 ortholog) [16], and polarized cell migration was previously shown to be impaired in JNCL patient fibroblasts [15]. Abnormalities in endocytosis, filopodia formation and polarized migration are in accordance with the observed Cdc42 cycling defect.

### CLN3 null cells have reduced plasma membrane localized ARHGAP21

**Figure 6:**
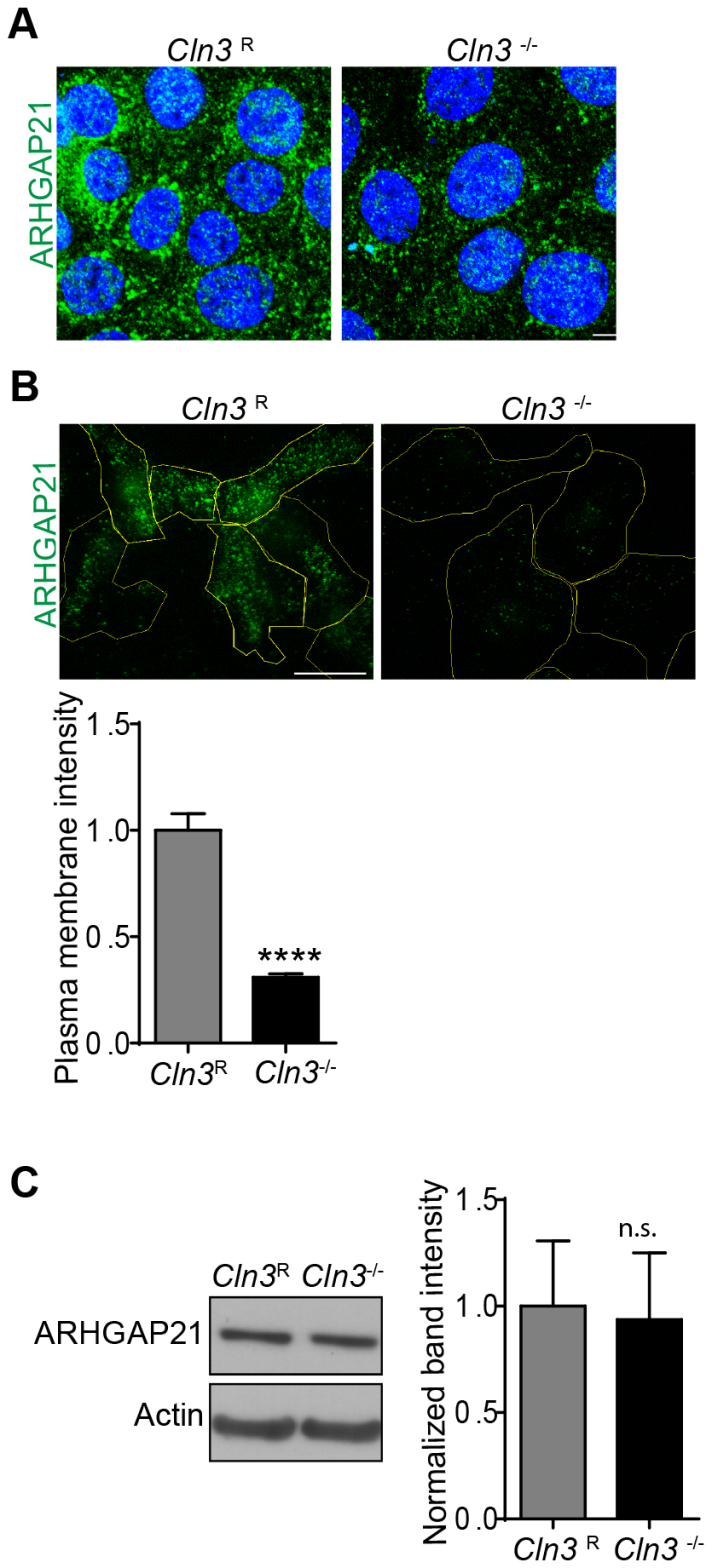
CLN3-null MBECs have reduced endogenous ARHGAP21 plasma membrane recruitment. Fixed MBECs were immuno-stained for endogenous ARHGAP21 (*green*). (A) ARHGAP21 was analyzed by confocal microscopy (cell nuclei, *blue*). (B) Basal membrane localization of ARHGAP21 was analyzed by TIRFM and quantified (≥70 cells per group). (C) Total endogenous ARHGAP21 protein levels were quantified by western blot, expressed as band intensity normalized to the actin loading control. Results represent the data from three independent experiments. Error bars are ± s.e.m. ((B,C) t-test, ****, p<0.0001, n.s. =  not significant). Scale bars are (A) 10 µm and (B) 25 µm.

For fluid-phase endocytosis, ARHGAP21 is recruited to the plasma membrane where it promotes the GTPase activity of Cdc42 [27] (Fig. 1). Given our finding of enhanced Cdc42-GTP, we wondered whether ARHGAP21 abundance or membrane recruitment is impaired in *Cln3* ^−/−^ MBECs. Using confocal microscopy, we observed a reduction in ARHGAP21 signal intensity in *Cln3* ^−/−^ MBECs (Fig. 6A) relative to normal cells. To test for changes in plasma membrane localization, total internal reflection fluorescence microscopy (TIRFM) was used, which showed that *Cln3* ^−/−^ MBECs have significantly reduced levels of plasma membrane localized ARHGAP21 (Fig. 6B). Western blot analysis showed equivalent ARHGAP21 protein levels in *Cln3* ^R^ and *Cln3* ^−/−^ MBECs (Fig. 6C), indicating that reduced ARHGAP21 TIRFM signal reflects a defect in membrane recruitment. Impaired recruitment of ARHGAP21 may thus underlie the amplified Cdc42-GTP and consequent reduction of fluid-phase endocytosis in the *Cln3* ^−/−^ MBECs.

### Overexpression of truncated ARHGAP21 reduces Cdc42-GTP and inhibits endocytosis

**Figure 7:**
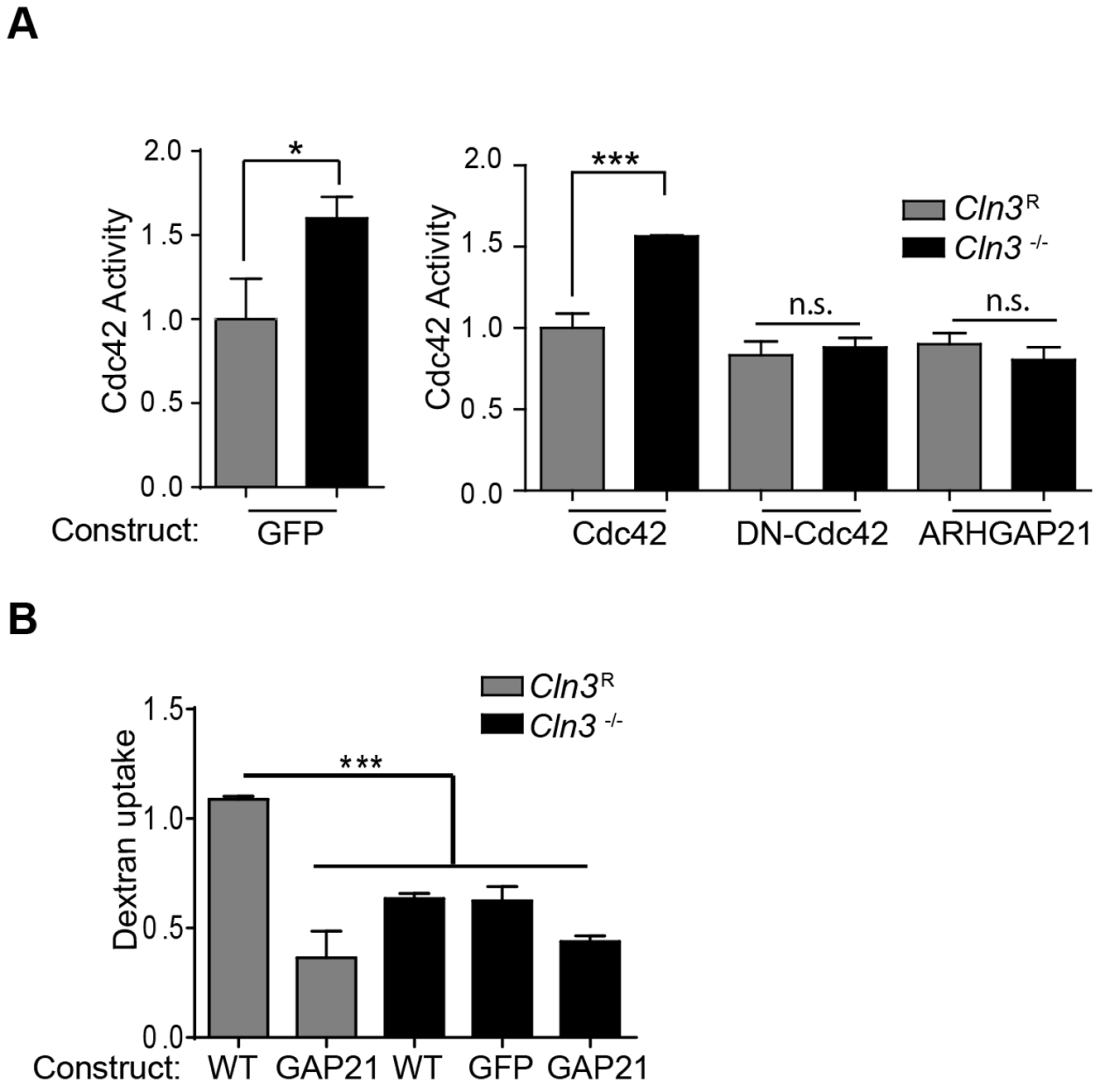
The impact of ARHGAP21 overexpression on endocytosis. (A) *Cln3* ^−/−^ and *Cln3^R^* MBECs were transfected with GFP, WT-Cdc42, dominant negative Cdc42 (DN-Cdc42), or ARHGAP21 expressing plasmids and Cdc42-GTP levels measured. (B) MBECs were transfected with GFP negative control, WT-Cdc42, or ARHGAP21(GAP21) constructs and rhodamine-conjugated dextran uptake was assessed as in Fig. 2. Results represent the mean of three independent experiments. Error bars are ± s.e.m. ((A left panel and B) t-test, (A right panel), ANOVA with multiple comparison test, *, p<0.05, ***, p<0.0001).

Initially we reasoned that transfection to enhance ARHGAP21 expression in CLN3-null MBECs might normalize Cdc42 activity and restore fluid phase endocytosis. However, while ARHGAP21 overexpression reduced Cdc42-GTP levels, (Fig. 7A), dextran uptake was not restored in CLN3-null cells (Fig. 7B & Fig. S2). These results indicate that ARHGAP21 overload creates an imbalance in favor of the inactive GDP bound state, with a resultant inhibitory effect (Fig.1, left panel). Similarly, DN-Cdc42 expression reduced Cdc42-GTP levels in *Cln3* ^−/−^ MBECs (Fig. 7A), while in contrast, WT-Cdc42 was without effect. Thus both DN-Cdc42 and ARHGAP21 potently limit Cdc42-GTP, to the point of inhibiting endocytosis, emphasizing that a fine balance of positive and negative modulation is required to regulate Cdc42 dynamics, with too much or too little impairing cell function (Fig. 1).

### *Cln3* ^−/−^ MBECs have reduced ARF1-GTP

**Figure 8:**
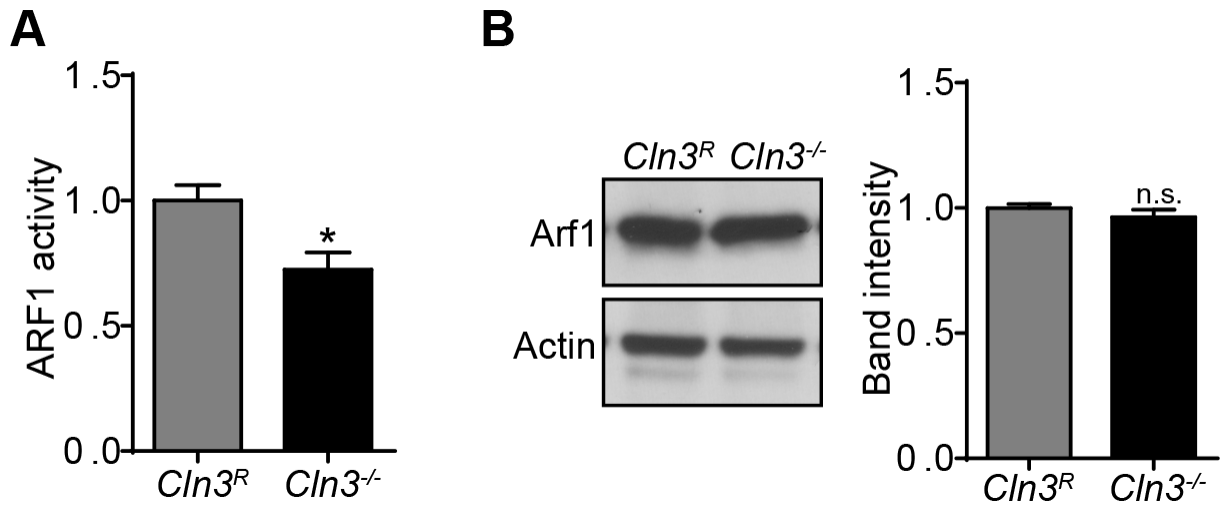
ARF1-GTP is reduced in CLN3-null MBECs. (A) ARF1-GTP levels were quantified in *Cln3* ^R^ and *Cln3* ^−/−^ MBEC lysates. (B) Total ARF1 protein levels were quantified in *Cln3* ^R^ and *Cln3* ^−/−^ MBEC lysates by western blot, expressed as band intensity normalized to the actin loading control. Data represent the mean of (A) five and (B) three independent experiments. Error bars ± s.e.m. (t-test, *, p<0.05, n.s. =  not significant).

ARF1-GTP promotes ARHGAP21 membrane recruitment and alterations in the GTP loaded state of ARF1 can inhibit fluid-phase endocytosis (Fig. S3) [27]. Therefore, we next tested whether endogenous ARF1 activity was altered in the absence of CLN3. Interestingly we found that *Cln3* ^−/−^ MBECs have lower ARF1-GTP compared to *Cln3^R^* MBECs (Fig. 8A). This finding provides an explanation for the reduced ARHGAP21 plasma membrane recruitment. These differences are not due to changes in ARF1 protein levels as evidenced by western blotting (Fig. 8B). Together our findings implicate compromised ARF1 activation as a proximal event of CLN3 deficiency, upstream of ARHGAP1 recruitment and endocytic defects.

## Discussion

Here we show for the first time that *Cln3* ^−/−^ MBECs have amplified Cdc42-GTP, increased phosphorylation of PAK-1 and LIMK recruitment, and impaired actin-dependent events including filopodia formation, cell migration, and fluid-phase endocytosis. Additionally, we show that ARHGAP21, a known regulator of Cdc42-mediated fluid-phase endocytosis, is mislocalized. We also demonstrate reduced levels of ARF1-GTP, a positive regulator of ARHGAP21 membrane recruitment. These data support a model where enhanced Cdc42 pathway activation is due to the loss of ARF1 activation (Fig. 1, right panel) in the setting of CLN3 deficiency.

Cell lines derived from patients or mice with CLN3 deficiency have impaired autophagy, vesicular trafficking, clathrin-independent endocytosis [13], and cell migration [15], all of which require actin-regulated steps. Consistent with these phenotypes, CLN3 has been reported to associate directly or indirectly with the actin regulatory proteins myosin-IIb, fodrin, and hook1 [15], [19], [30]. Our data implicate the small GTPase Cdc42 as a common link.

In addition to Cdc42, other Rho GTPase family members can regulate actin-dependent processes, and may act coordinately to regulate cytoskeletal changes that affect cell shape and migration [31]. While it is clear from our study that CLN3 null cells display ARF1-Cdc42 pathway defects consistent with endocytic, filopodia and cell migration defects, it remains to be determined whether CLN3 loss also impacts the activity of Rho GTPases, further contributing to actin-related phenotypes. Here we used P-PAK1 and LIMK membrane as indicators of Cdc42 activity, but these proteins are also regulated by other Rho GTPases [32], [33]. Structures such a filopodia can be the product of Cdc42 and Rac1 regulation [31] as these GTPases cross activate one another [34]. Future investigation will address whether activation of other Rho GTPases is aberrant.

The filopodia defects are novel observations, and may play a part in functional decline in JNCL brain. In addition to their role as environmental sensors, filopodia are the foundation for many integrin-matrix connections [35]. Interestingly, *in vivo*, endothelial cells with more filopodia have impaired tight junctions [36]. It is possible that the filopodia phenotype contributes to BBB destabilization. Interestingly, modulation of both PAK1 and LIMK are important regulators of BBB permeability and when dysfunctional can contribute to neurodegeneration [37], [38].

Under quiescent conditions *in vivo*, fluid-phase endocytic activity in brain endothelial cells is low [39]. During stress, fluid-phase endocytosis is upregulated [40], modifying the plasma membrane composition and BBB permeability [41]. As Cdc42 is important for adhesion and fluid-phase endocytosis, *Cln3* ^−/−^ mice and JNCL patients may display BBB dysregulation when stressed, such as during an infection or as neurons succumb to disease.

Interestingly, ARF1 binding to ARHGAP21 negatively regulates Cdc42 activity at both the plasma membrane [27] and Golgi [42], [43]. Cdc42 activity at the Golgi may also be impaired. Indeed, we find a dispersed ARHGAP21 staining pattern in CLN3-null MBECs that is similar to the ARHGAP21 pattern seen during overexpression of a dominant negative ARF1 [28]. Previous observations of a fractionated Golgi morphology [44], [45] and impaired anterograde [46] and retrograde [47] transport at the Golgi in CLN3 null cells, are consistent with defects in ARF1. In a separate study, we found that CLN3-null MBECs display impaired anterograde trafficking of caveolin-1 to the plasma membrane, with consequent reduction in caveolae formation [13]. Interestingly, in addition to its role in fluid-phase endocytosis [27], ARF1 promotes caveolin-1 trafficking from the Golgi [48].

We speculate that CLN3 influences ARF1 dependent functions at both the plasma membrane and Golgi apparatus. Interestingly, one of the proteins identified by mass spectrometry analysis of CLN3-pulled down proteins was GBF1 [49]. GBF1 is a well-described Arf1-GEF critical to ARF1 activation and vesicular transport at the Golgi [50], [51] and plasma membrane [52], [53]. We predict that CLN3 may participate in GBF1 recruitment. Alternatively, CLN3 may affect protein localization indirectly by influencing the lipid microenvironment. Loss of CLN3 results in increased plasma membrane fluidity [13] and CLN3 has been implicated in lipid interaction/transport and metabolism [3]. Notably, proposed scaffolding and membrane effects are not necessarily exclusive; CLN3 may interact with key proteins and trigger dynamic lipid modifications at sites of protein assemblage.

In summary, we report elevated Cdc42 activation as a novel phenotype in CLN3-deficient MBECs, which may be integral to multiple actin-dependent cellular functions. Endothelial and neuronal defects in Cdc42 activity may contribute to BBB defects and brain pathology in JNCL patients. We provide evidence that reduced ARF1 activity and subsequent impaired membrane recruitment of ARHGAP21 is the basis for the defective Cdc42 cycling. Thus, therapeutics to improve ARF1 activity, the trafficking of ARHGAP21, or Cdc42-GTPase activity may be effective treatments for JNCL. As drugs are being developed for other GTPases [54], ARF1 or Cdc42 are potentially suitable targets for therapeutic development for JNCL, a currently untreatable, fatal neurodegenerative disease.

## Materials and Methods

### Mice

All animal experiments were approved by the University of Iowa animal care and use committee and conducted in accordance with institutional and federal guidelines. In this study C57BL/6J (WT) and *Cln3* ^lacZ/lacZ^ (*Cln3* ^−/−^) mice [10] on the C57BL/6J background were used.

### Antibodies

Cdc42 1∶1000 (Cell Signaling Technology, Danvers, MA USA), LIMK H-84 1∶500 (Santa Cruz, Dallas, TX USA), PAK1 1∶700 (Epitomics, Burlingame, CA USA), P-PAK1 1∶1500 (Epitomics), β-actin 1∶5,000 (Sigma, St. Louis, MO USA), TFNR 1 µg/ml (Invitrogen, Grand Island, NY USA), β-catenin (Abcam, Cambridge, MA USA), GFP 1∶1000 (Abcam ab290), and ARHGAP21 1∶300 (Sigma, Santa Cruz; H-300), ARF1 (abcam EPR443), and Acti-stain-488 (Cytoskeleton, Denver, CO USA).

### MBEC collection and cell lines

MBECs from WT and *Cln3* ^lacZ/lacZ^ mice were collected and cultured as described previously [55]. To minimize cell loss we employed puromycin selection as described previously [56]. To provide sufficient cells for the many cell based assays used here, we immortalized *Cln3* ^lacZ/lacZ^ MBECs with retrovirus strategies to create the immortalized cell line *Cln3* ^−/−^ [13]. To create a CLN3 restored control cell line, *Cln3* ^−/−^ cells were transduced with FIV-*Cln3*, which stably restored *Cln3* expression (*Cln3* ^R^). The immortalized *Cln3* ^−/−^ and *Cln3* ^R^ lines display brain endothelial markers and behave similar to respective primary CLN3-null and WT MBEC cultures in functional assays [13].

### Fluid-phase endocytosis

Cells were incubated with Hoechst 33342 (Pierce Biotechnology, Rockford, IL USA) for 1 hour, then Alexa Fluor 488 or Rhodamine 10,000 MW dextran (Invitrogen, Grand Island, NY USA) was added to cell culture media to a final concentration of 0.5 and 0.25 mg/ml and cells incubated for 20 minutes. Media containing dextran was aspirated, and cells were washed briefly 3X with 37°C PBS, removing excess dextran. To stop dextran intake and trafficking, cells were fixed in 3.7% paraformaldehyde at 37°C for 10 minutes. This confined dextran signal to the plasma membrane and endocytic vesicles. In Fig. 2 A, B extracellular and plasma membrane fluorescence was quenched by adding membrane impermeable 200 mM Red-40 [57] (Spectrum, Gardena, CA USA). Images to assess internalized dextran were taken with an Olympus IX81 microscope and uptake was quantified with ImageJ using the same settings for each experimental group. Average intensity/cell area was determined for each field. A minimum of 6 fields were taken per experiment. Significance was tested by Student's t-test. Images were assembled in Adobe Photoshop and levels, contrast, or brightness adjusted on the entire experimental group if necessary.

### Cdc42 and ARF1 activity assay

Total cell lysates were collected and flash frozen in liquid nitrogen to minimize GTP hydrolysis. Cdc42-GTP and ARF1-GTP were quantified by the *G-Lisa* ^®^ kit from Cytoskeleton Inc. (Denver, CO USA) as per the manufactures instructions except cells were not serum starved. A concentration of 0.8 mg/ml (Cdc42) and 1 mg/ml (ARF1) of cell lysate was added to the ELISA. Data were normalized to Cdc42 activity in wild type or *Cln3* ^R^ cells and significance was determined by Student's t-test.

### Cell lysis for Western blot

Lysis buffer containing 0.5% Triton x-100 with protease and phosphatase inhibitors (Roche, Madison, WI USA) was added to MBECs culture plates and incubated on a rocking platform at 4°C for 10 minutes. After removing cells using a cell scraper, nuclei were briefly sonicated and pelleted by 1,000×g centrifugation and the supernatant was collected. Protein concentrations were determined by the DC protein assay (Bio-Rad, Hercules, CA USA), and equilibrated. Samples were loaded and run on (4–12% Tris-Bis NuPAGE) gels, and transferred by western blot to PVDF membranes. Membranes were blocked with 5% BSA, and immuno-blotted for Cdc42, PAK1, P-PAK1, LIMK, β-actin, TRFNR, ARHGAP21, ARF1, GFP, and β-catenin using standard methods. Blots were incubated with the primary antibodies overnight at 4°C and secondary antibodies for 1 hour at room temperature. Immunoreactive bands were quantified by densitometry using Quantity One software with the VersaDoc imaging system (Bio-Rad). Band intensities were normalized to a house-keeping protein band (β-actin, TRFNR, or β-catenin) in the same lane and significance was determined by t-test.

### Membrane enrichment

Cell culture medium was aspirated and membrane prep solution (0.25 M Sucrose, 50 mM MOPS, 2 mM EDTA, 2 mM EGTA pH 7.4) was added to MBECs, whereupon cells were immediately removed with a cell scraper. After brief sonication, nuclei were removed by a 2,500×g centrifugation for 5 minutes. Supernatant were collected and spun at 200,000×g for 1 hour to enrich for cell membranes. Membranes were resuspended in RIPA buffer and equal amounts of protein loaded onto gels for SDS-PAGE, western blot, and band quantification as above.

### SEM and filopodia quantification

Sub-confluent cells were fixed with glutaraldehyde, dehydrated overnight, and coated with gold and platinum. The following day, filopodia were visualized with a Hitachi S4800 (Dallas, TX USA) scanning electron microscope. High magnification images were compiled with Adobe Photoshop and the multimeasure tool in ImageJ was used to measure cell membrane and filopodia length. Filopodia ≥2 µm were counted and measured, and filopodia per length of membrane determined. For each image the sum of filopodia lengths was divided by the number of filopodia to calculate the average filopodia length. Significance was assessed by a Student's t-test.

### Scratch Assay

A pipette tip was used to scratch a confluent monolayer of cells. Cellular debris from the scratch was removed by a PBS wash. Serial images were taken by an Olympus IX81 (Center Valley, PA USA) live cell microscope overnight. ImageJ was used to calculate the percent area remaining at each time point. Significance was assessed by 2-way ANOVA with Bonferroni post hoc-test.

### MBEC transfection and constructs

Subconfluent cells were transfected using the lipid-based Lipofectamine LTX (Invitrogen, Grand Island, NY USA) applying half of the manufactures suggested concentrations for endothelial cells. Experimental analysis was conducted the following day. The following constructs were used: hrGFP (empty vector control), GFP-Cdc42 (WT), dominant negative GFP-Cdc42 (T17N), constitutively active GFP-Cdc42 (Q61L) [26], GFP-ARF1 (WT), dominant negative GFP-ARF1 (T31N), constitutively active GFP-ARF1 (Q71L) [58],GFP-ARHGAP21 (amino acids 855–1346) [28], [59].

### Confocal microscopy

After aspiration of cell culture media, cells were briefly rinsed in PBS (37°C), fixed in 4% PFA (37°C), and permeabilized, and blocked with Image-iT^®^ FX Signal Enhancer (Life Technologies, Grand Island, NY USA). After an overnight incubation with Anti-ARHGAP21 (Santa Cruz, Dallas, TX USA) and 1 hour of secondary Ab (1∶2000) cell were imaged on a Zeiss LSM710 (Thornwood, NY USA) microscope.

### TIRFM

The Image-iT FX^®^ signal enhancer protocol (Invitrogen) was followed for cell fixation, permeabilization, and prevention of nonspecific binding. Cells were incubated with ARHGAP21 primary antibody 1∶300 (Sigma) overnight and imaged with a Leica AM TIRF Imaging System (Leica Microsystems, Philadelphia PA, USA). Images were taken with DIC and TIRF (90 nm penetration depth) channels. LAS AF software (Leica Microsystems) was used to quantify cell area in the DIC channel and TIRF intensity. The following equation was used to quantify images:
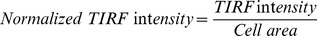



## Supporting Information

Figure S1 **Altered actin structures in CLN3-null MBECs.** Subconfluent MBECs were fixed and stained with Acti-stain 488 which stains F-actin. Z-stacks were taken by confocal microscopy and ImageJ used to compile images.
(TIF)

Figure S2 **Overexpression of ARHGAP21 reduces fluid-phase uptake in CLN3-null MBECs.** A) CLN3-null MBECs were transfected with WT-Cdc42-GTP or ARHGAP21-GFP and fluid-phase endocytosis was quantified. Of note, though ARHGAP21 is GFP-tagged, GFP fluorescence is compromised in the context of the fusion protein, but transfected cells could be visualized by increasing exposure (transfected cells outlined with white dashed lines). Western blot analysis of transfected cells and immuno-blotted with anti-GFP antibody confirmed ARHGAP-GFP expression. Actin was used as a loading control. Scale bar  = 10 µm.
(TIF)

Figure S3 **ARF1 is an upstream regulator of fluid-phase endocytosis.** *Cln3* ^R^ MBECs were transfected with GFP (Negative control), WT-ARF1-GFP, dominant negative (DN)-ARF1-GFP, or constitutively active (CA)-ARF1-GFP constructs (*green*). Transfected cells were imaged and endocytosis of Rhodamine conjugated dextran (*red*) was quantified as in Fig. 2. Data represent the mean of three independent experiments. Error bars ± s.e.m. (1-way ANOVA with Tukey post-hoc, *, p<0.05, n.s. =  not significant). Scale bar represents 10 µm and dashed lines represent the outline of transfected cells.
(TIF)
