# Supplementary figures and images for: CLN3 Deficient Cells Display Defects in the ARF1-Cdc42 Pathway and Actin-Dependent Events
Source: PLoS One. 2014 May 2;9(5):e96647. doi: 10.1371/journal.pone.0096647 (PMC4008583; doi:10.1371/journal.pone.0096647)

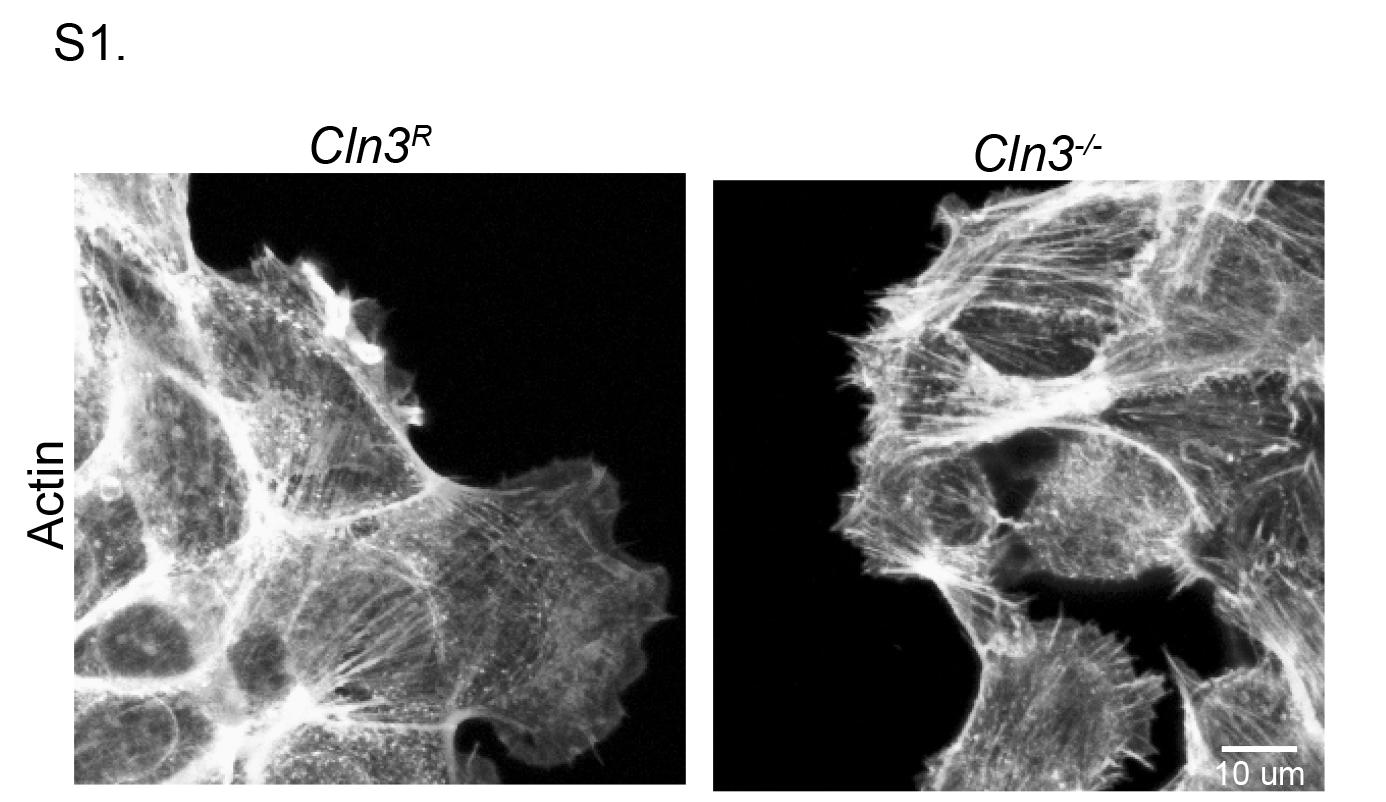

Supplement: Figure S1 — Altered actin structures in CLN3-null MBECs. Subconfluent MBECs were fixed and stained with Acti-stain 488 which stains F-actin. Z-stacks were taken by confocal microscopy and ImageJ used to compile images. (TIF) [file pone.0096647.s001.tif]

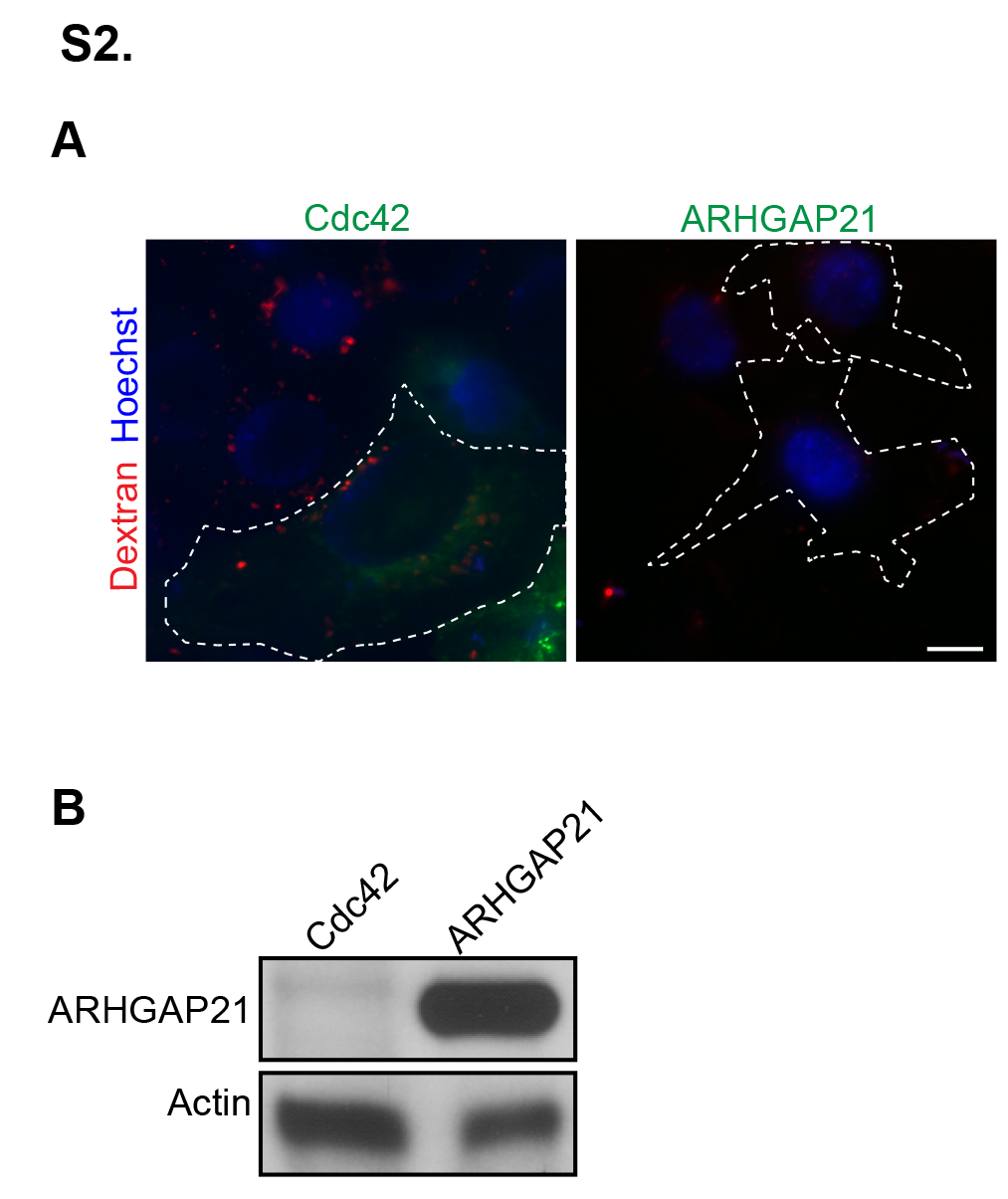

Supplement: Figure S2 — Overexpression of ARHGAP21 reduces fluid-phase uptake in CLN3-null MBECs. A) CLN3-null MBECs were transfected with WT-Cdc42-GTP or ARHGAP21-GFP and fluid-phase endocytosis was quantified. Of note, though ARHGAP21 is GFP-tagged, GFP fluorescence is compromised in the context of the fusion protein, but transfected cells could be visualized by increasing exposure (transfected cells outlined with white dashed lines). Western blot analysis of transfected cells and immuno-blotted with anti-GFP antibody confirmed ARHGAP-GFP expression. Actin was used as a loading control. Scale bar = 10 µm. (TIF) [file pone.0096647.s002.tif]

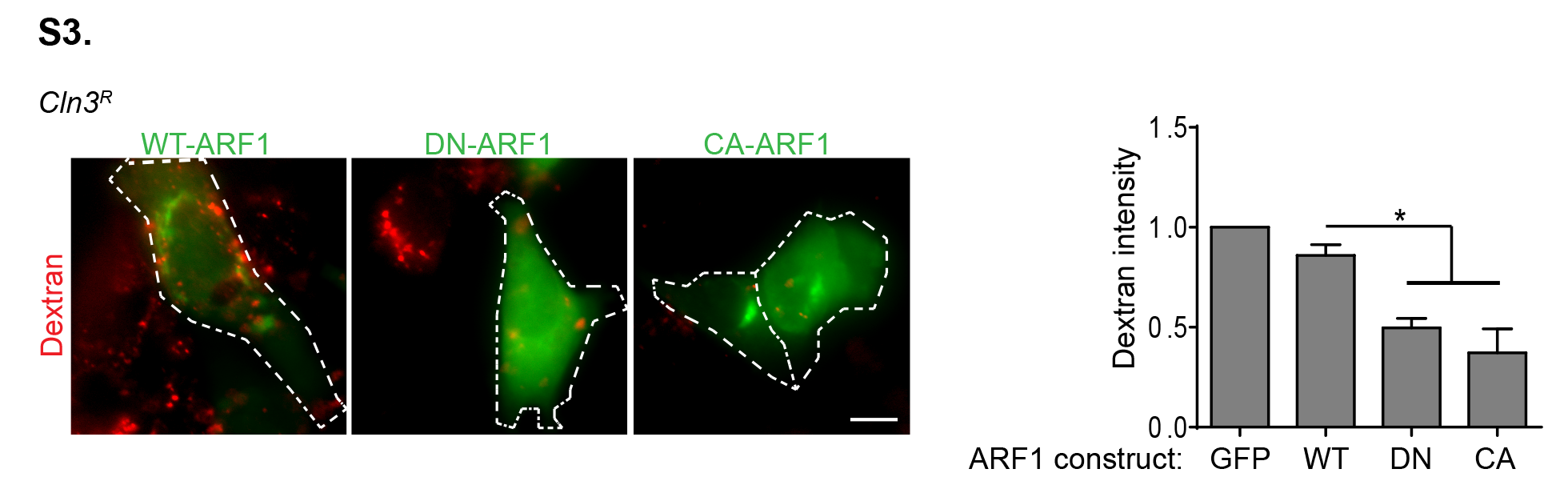

Supplement: Figure S3 — ARF1 is an upstream regulator of fluid-phase endocytosis. Cln3 R MBECs were transfected with GFP (Negative control), WT-ARF1-GFP, dominant negative (DN)-ARF1-GFP, or constitutively active (CA)-ARF1-GFP constructs (green). Transfected cells were imaged and endocytosis of Rhodamine conjugated dextran (red) was quantified as in Fig. 2. Data represent the mean of three independent experiments. Error bars ± s.e.m. (1-way ANOVA with Tukey post-hoc, *, p<0.05, n.s. = not significant). Scale bar represents 10 µm and dashed lines represent the outline of transfected cells. (TIF) [file pone.0096647.s003.tif]
